# Prevalence and antibiotic resistance of *Campylobacter coli* isolated from broiler farms in the Marrakesh Safi region, Morocco

**DOI:** 10.14202/vetworld.2020.1892-1897

**Published:** 2020-09-16

**Authors:** Raja Asmai, Bouchra Karraouan, Khadija Es-Soucratti, Houda En-Nassiri, Brahim Bouchrif, Hakim Karib, Réda Triqui

**Affiliations:** 1 Unité HIDAOA, Departement de Pathologie et Santé Publique Vétérinaire, Institut Agronomique et Vétérinaire Hassan II, B.P. 6202, Rabat-Instituts, Rabat, Morocco; 2 1, Place Louis Pasteur 20360, Casablanca, Morocco; 3 Department of Biology, Ain Chock Faculty of Sciences, Hassan II University, Casablanca, Morocco

**Keywords:** *Campylobacter coli*, microbial resistance, Morocco poultry, prevalence

## Abstract

**Background and Aim:**

Campylobacteriosis is a common foodborne disease epidemiologically linked to the consumption of poultry products. However, other sources, such as raw or contaminated milk, contaminated water or ice, contact with infected livestock, and pets, are reported. This study aimed to evaluate the prevalence and resistance to microbial resistance of *Campylobacter coli* in broiler farms in the region of Marrakesh Safi, Morocco.

**Materials and Methods:**

The study was conducted between May and December 2017 and involved 35 broiler farms. One hundred and five cloacal swabs were collected from the eight provinces in the region of Marrakesh Safi, Morocco. Bacteriology method NM ISO/TS 10272-3: 2013 was used to isolate and identify *Campylobacter* spp. Molecular identification (polymerase chain reaction) was used for confirmation. A disk diffusion method on Mueller-Hinton agar was used for susceptibility testing. Five antibiotic agents, including first-line drugs, were evaluated.

**Results:**

Among 105 samples, 71.4% (75/105) were positive for *Campylobacter* spp. test and 56% (42/75) of isolates belonged to the species *coli*. Susceptibility profiles showed that 95.2% of *C. coli* strains were resistant to ampicillin, 92.8% to erythromycin and tetracycline, 85.7% to ciprofloxacin, and 7.1% to gentamicin.

**Conclusion:**

This study underlines the need to strengthen implementation of specific control procedures to decrease contamination of poultry meat with *Campylobacter* spp. and to reduce the use of antibiotics in the poultry sector.

## Introduction

Thermophilic campylobacters are the most common bacterial cause of diarrhea in humans worldwide. *Campylobacter jejuni* and *Campylobacter coli* together account for more than 95% of *Campylobacter* infections in humans [1]. The acute phase of infection is characterized by diarrhea, fever, abdominal cramps, and vomiting. Such infections are the main cause for the development of Guillain-Barré syndrome, reactive arthritis, and irritable bowel syndrome, which all occur as sequelae after the acute phase [2].

The WHO estimated that *Campylobacter* causes 37.600 deaths/year worldwide [3]. This infection surpassed *Salmonella* several years ago as a cause of death and continues to cause a significant economic burden [4].

According to the Moroccan Interprofessional Federation of the Poultry Sector, poultry production in Morocco is the most dynamic agricultural activity with an average annual increase of 6% for broiler meat and 5.7% for egg production. More than a third of animal protein in the Moroccan diet is provided by the poultry sector. In 2016, this sector generated 2.8 billion and made investments of 1.3 billion dollars. It provides 120.000 direct jobs and 280.000 indirect jobs, especially in marketing and distribution [5].

The transmission of *Campylobacter* from animal food sources to humans is not uncommon, due to the growing demand for meat and other products from livestock. Increasing antibiotic resistance in *Campylobacter* strains from animal sources is well reported globally [6]. Worldwide, inappropriate use of antimicrobials in animal husbandry is a major concern because of the associated development of resistant and even multidrug-resistant (MDR) bacteria. Antibiotics in poultry production systems are widely used to prevent, control, and treat bacterial infections as well as stimulate growth in many countries [7].

This study aimed to determine the prevalence and antimicrobial resistance of *Campylobacter* in broiler farms in the Marrakesh Safi region, Morocco. Such information may help establish effective surveillance programs and control measures for *Campylobacter* in the Moroccan poultry industry.

## Materials and Methods

### Ethical approval

This study did not use live animals, so ethical approval was not needed.

### Study area

The study was conducted in the Marrakesh Safi region, Morocco, which covers an area of 41.404 km^2^ and has 4.521 million inhabitants [8]. There are several poultry type activities in the mentioned area [9].

### Sampling

The study was conducted between May and December 2017 and involved 35 broiler farms of the Marrakesh Safi area. Flock ranged from 5000 to 10,000 birds aged 2-4 weeks. A total of 105 samples (7/farm) were collected by a qualified veterinarian inspector routinely involved in cloacal swabs sampling for national surveillance programs. Sampling was performed on a regular basis for the purpose of obtaining the same number of samples for both warm and cold sampling periods (n=52). Samples were soaked in a transport medium (Preston Broth) to prevent drying and dilute traces of urine that could lower the level of reliability of detection, according to indications from the OIE. All samples were transported in cold condition (4°C) within 3 h to the food microbiology laboratory of the Institut Pasteur du Maroc and analyzed the same day. This laboratory is accredited according to standard NM ISO/CEI 17025: 2018-Ref AL86/2016.

### Isolation and identification

Isolation of *Campylobacter* spp. used the horizontal method for detection and enumeration (ISO 10272-3:2013). Two milliliters of cloacal swab slurry was transferred to 9 mL of Preston enrichment broth (CM 0067 Oxoid, Oxoid LTD., Basingstoke, Hampshire, UK) containing *Campylobacter* growth factor (SR 0232E Oxoid, Oxoid Ltd., Basingstoke, Hampshire, UK) and 7% (v/v) defibrinated sheep blood; and incubated for 24 h at 42°C in anaerobic jars containing packet generators of microaerophilic atmosphere (5% oxygen, 10% carbon dioxide, and 85% nitrogen) type CAMPYGen (CN0025A Oxoid, Basingstoke, Hampshire, UK).

After enrichment, each sample was directly streaked onto *Campylobacter* Selective Agar (Base) (Oxoid Ltd., Basingstoke, England) containing 5% fresh sterile defibrinated sheep blood and *Campylobacter* supplement III (Sigma, St. Louis, MO, USA) for primary isolation.

Incubation continued for 72 h at 37°C, again using anaerobic jars. *Campylobacter* spp. colonies from each agar plate were streaked on blood Colombia agar plates and incubated under anaerobic conditions (10% CO_2_, 5% O_2_, and 85% N_2_) for 24 h at 37°C. All isolates were confirmed as *Campylobacter* spp. by biochemical tests (oxidase, catalase, and ­hippurate hydrolysis), motility, microscopic examination, Gram staining, and by conventional polymerase chain reaction (PCR) assay, as described by Hayachi [10]. All isolates were stored at −80°C in Brucella broth (Thermo Fisher Scientific, Milan, Italy) until used.

### *Campylobacter* genotyping

DNA was extracted from agar plates using InstaGene1matrix (Bio-Rad, California, USA) following the manufacturer’s instructions. DNA extracts were stored at −20°C until used, primers were custom synthesized to amplify members of *C. coli* for the molecular identification [11].

PCR used a final volume of 25 μL containing 4 μL of extracted DNA, 12.5 μL of Taq DNA polymerase (BioMix™ Red, a Meridian Life Science, Germany), 0.5 μL MgCl_2_ (25 mM), and 0.2 μL of each primer (25 mM). Amplification conditions were 95°C for 5 min, followed by 35 cycles of denaturation at 95°C for 1 min, annealing at 48°C for 1 min, and extension at 72°C for 1 min and final extension at 72°C for 10 min. Amplicons were separated on 2% agarose gels and stained with ethidium bromide. DNA bands were visualized under ultraviolet light.

### Antibiotic susceptibility

Isolates were screened for susceptibility to five antibiotics on Mueller-Hinton agar (Oxoid, Basingstoke, Hampshire, United Kingdom) by disk diffusion, as described in the “Comité Français de l’Antibiogramme-Société Française de Microbiologie” (CA-SFM/EUCAST V2 Mai 2019) guidelines. The antibiotics used were ampicillin (10 μg), tetracycline (30 μg), ciprofloxacin (5 μg), erythromycin (15 μg), and gentamycin (10 μg). Plates were incubated at 35±2°C for 24-48 h, and diameters of the inhibition zones were measured using a caliper. The measurements were compared with the zone size interpretative chart furnished by CA-SFM/EUCAST V2 May 2019. Zones were graded as susceptible, intermediate resistance, considered in the statistical analysis as resistance, and resistance.

### Sample size and statistical power

The sample size was determined based on an expected prevalence of 88% (previous studies of prevalence in Spain and Portugal) [12] and acceptable error of 5% and 95% confidence giving a required sample size of 105 samples, as:

N = [(Z_alpha)^2 * p * (1 − p) ]/L where N is the number of broilers to be sampled, Z-alpha is 1 - alpha/2 percentiles of a standard normal distribution to obtain 1-alpha 100% confidence level, p is the a-priori estimate of the prevalence, and L is the required precision of the estimate. We computed the effective sample size and statistical power using the PWR Package of R software (https://cran.r-project.org/view=ClinicalTrials). With 105 samples, the expected power reaches 100%.

### Statistical analysis

Proportions of positive samples detected in 15 farms were analyzed. Categorical variables are presented as proportions. Continuous variables are presented as mean and standard deviations if normally distributed or median and interquartile range if non-normally distributed.

Descriptive statistics were used to portray the study population at baseline. The comparison of means among two study groups (positive Campylo test vs. negative) used analysis of variance and Wilcoxon or Kruskal–Wallis tests, as appropriate. Comparisons used Chi-square or Fisher’s exact tests, as appropriate, with Bonferroni correction. p≤0.05 is considered statistically significant. A logistic regression model with a backward elimination procedure was employed to identify factors associated with positive Campylo tests.

## Results and Discussion

Our results highlighted the prevalence of *Campylobacter* spp., more specifically *C. coli*, in broiler farms along with their antibiotic resistance profiles. One hundred and five samples from broilers aged 2-4 weeks were analyzed, and 71.4% (75/105) were positive for *Campylobacter* spp. and the remainder (30/105) negative. All the *Campylobacter* spp. strains were screened by conventional PCR using the following primers: CC7723 (Forward): 5‘ATATTTCCAAGCGCTACTCCCC 3’ and CC7724 (Reverse): 5’CAGGCAGTGTATAGTCATGGG3’ [11]. About 56% (42/75) of strains display the same band size (258 bp) as the reference strain, *C. coli* ATCC 4378.

**Figure-1:**
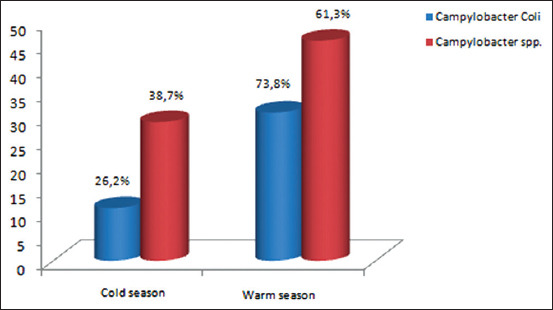
Prevalence of *Campylobacter* spp. and *Campylobacter coil* isolated in different seasons.

**Figure-2:**
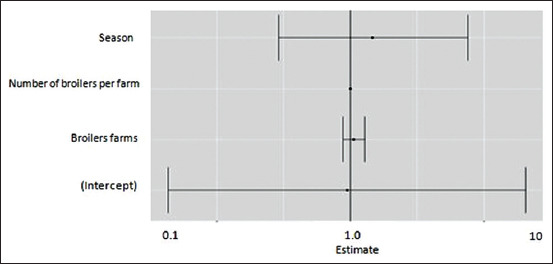
Odds ratios for the three predictors: Season, broiler farms, and number of broilers.

We compared samples taken during the cold period (from September to December) and samples collected in the warm period (from May to August). Our results are in agreement with a higher prevalence in the warm period (Figure-1). Results of logistic regression modeling are presented as odds ratios (ORs) and 95% confidence intervals (Figure-2) and (Table-1). ORs were adjusted for farms, which are related to Campylo testing. Adjusting did not change the results. All analyses were performed with R software version 3.3.2 (https://cran.r-project.org/bin/windows/base/old/3.2.2/). ORs for three predictors “­season, broiler farm, and number of broilers per farm are >1.” A *Campylobacter* positive test is more likely to occur as predictors increase.

**Table-1:** Results of logistic regression modeling with odds ratios.

Characteristic	OR	95% CL
Season	1.46	0.29, 7.57
Broilers farms	1.06	0.88, 1.29
Number of broilers per farm	1.00	1.00, 1.00

OR=Odds ratio, Cl=Confidence interval

Differences in prevalence between the warm and cold periods were statistically significant according to the Chi-square test (p<0.05). *Campylobacter* spp. prevalence in our investigation area was close to that of European baseline studies (71.2%). This prevalence varied from 2% in Estonia to 100% in Luxembourg [13]. Our results were much lower than those found in Algeria (98%) [14], Spain (88%), Portugal (82%), and Malta (96.3%) [12].

Nevertheless, prevalence identified present study is substantially higher than the prevalence reported in European Nordic countries, such as Sweden (13.2%), Finland (3.9%), and Denmark (19%) [12]. Most North European countries have actively implemented successful strategies to control *Campylobacter* and have consequently achieved a lower prevalence of *Campylobacter* in broiler flocks [12].

The highest positive percentage of *Campylobacter* in different areas of Marrakesh Safi in Morocco was encountered in poultry sampled in the warm season (50%) and a lower prevalence in the cold season (15%). No significant differences in prevalence were found for region distribution since the samples were all collected in the same geographic region. Seasonal variation in *Campylobacter* prevalence in broilers, with a peak in the summer has been previously reported from several countries in North Europe [12].

The reason for the association of higher *Campylobacter* prevalence and season is not known but appears to be temperature related [14]. Seasonality could be related to the abundance of flies in the summer, which act as mechanical vectors [15]. During summer, ventilation and water consumption also increase because of the higher temperatures and temperature could also affect environmental sources of *Campylobacter* spp. to which broiler chickens are exposed. Further, a close association between such environmental contamination and the weather was found [16]. Thus, the high prevalence of *Campylobacter* is related to season rather than to other factors. In fact, in spring and summer, the weather in Morocco is warm and humid, and the highest heat index values are observed in summer. The same data are reported in the literature and are supported by the seasonality of disease in humans, with a peak incidence always observed during warm and humid seasons [17].

Low winter prevalence of human disease appears important to seasonality and likely indicates differences in the importance of risk factors at various times of the year.

### Antimicrobial resistance

All isolates (n=42) were tested for antimicrobial susceptibility. All strains were resistant to one or more antimicrobial agents. Percentage of strains resistant to antimicrobial resistance was, in decreasing order, ampicillin (95.2%), erythromycin and tetracycline (92.8%), and ciprofloxacin (85.7%). The lowest resistance was observed for gentamycin (7.1%). MDR is defined as acquired non-susceptibility to at least one agent in three or more antimicrobial categories [18]. Among the resistant isolates, 42 (100%) were resistant to more than 2 classes of antibiotics. Forty isolates (95%) displayed drug resistance to ≥3 drugs (Table-2).

**Table-2:** Resistance pattern profiles of isolated *Campylobacter coli* strains.

Associated resistances to	Resistance pattern profiles	Number of strain	Number of total (%)
Two antibiotics	TE, E	2	2 (4.8)
Three antibiotics	AM, GM, CIP	3	3 (7.1)
	AM, TE, E	4	4 (9.5)
Four antibiotics	AM,TE, E, CIP	33	33 (78.6)

AM=Ampicillin, E=Erythromycin, G=Gentamicin, TE=Tetracycline, CIP=Ciprofloxacin

Antibiotic resistance of *Campylobacter* spp. is a persistent issue in both veterinary and human medicine because of the indiscriminate use of antibiotics in therapy or as growth promoters. More than 77% of isolates were resistant to fluoroquinolones (ciprofloxacin). In contrast, resistance was much lower in 2007 as reported by Jouahri (44.4%) [19]. High resistance is also reported in Algeria (83.7%) [14]. Increased resistance to ciprofloxacin can be explained by the introduction of enrofloxacin, a molecule closely related to ciprofloxacin, to poultry farming in Morocco in 1990, and its wide use for the treatment of *Escherichia coli* infections [18].

Most *Campylobacter* infections are self-limiting, but antimicrobial treatment is necessary for some severe and prolonged cases [20]. Fluoroquinolones and macrolides are usually administered to treat human campylobacteriosis [21]. According to a WHO report, no fluoroquinolone-resistant isolates are detected in Norway [22], and only 9.4% of *Campylobacter* strains are resistant to the drug in Granada, Spain [23].

Among strains of *C. coli* analyzed in the present study, 95% were resistant to erythromycin. The same average level was found in a study carried out recently in Casablanca, Morocco [24]. Such a level is somewhat alarming in comparison to previous results from the Oujda area of East Morocco [19]. Data for *C. jejuni* and *C. coli* included in the European Food Safety Authority (EFSA) report indicate resistance to erythromycin was about 6%, but a recent increasing trend has been observed [12].

Tetracycline, previously listed as an alternative treatment for *Campylobacter* gastroenteritis, is widely used therapeutically for livestock and poultry. According to the EFSA report, resistance in *Campylobacter* spp. isolated from broilers in 2007 to this antimicrobial was 76% for *C. coli* [12]. In the present study, the observed resistance incidence is similar.

Our actual resistance frequencies to ampicillin (95.2%) and gentamycin (7.1%) are comparable to those of most European countries. In Italy, for example, 27.9% isolates were sensitive to gentamycin and 20.9% to both ciprofloxacin and erythromycin, respectively [25]. In North African countries such as Algeria and Tunisia, the percentage of resistance to amoxicillin and clavulanic acid is much lower (27%) compared to our current results. These discrepant results may be related to the intrinsic antibacterial activity of clavulanic acid and inhibition of β-lactamases [26].

Finally, where the use of fluoroquinolones in poultry production is prohibited, such as Australia and North European countries, few resistant *Campylobacter* isolates are found in chicken or humans. In Australia, 15.4% were resistant to ampicillin, 5.1% to tetracycline, and 13.7% to ciprofloxacin [27]. In Canada, a high frequency of resistance to tetracycline was observed (77%), but the frequency of resistance to quinolones and macrolides was low [28]. To assess the impact of an industry-wide policy change in antimicrobial use in Canada, antimicrobial resistance of *Campylobacter* spp. was compared between 2003 and 2015. Ciprofloxacin-resistant *Campylobacter* spp. significantly increased (33%), along with a rise of 7% resistance for telithromycin, azithromycin, clindamycin, and erythromycin [29].

## Conclusion

High contamination levels of broilers (71.4%) in Morocco by *Campylobacter* spp. are evidenced by the results of the present investigation, with a specific prevalence of 56% for *C. coli*. A seasonal effect on prevalence was also observed, with a higher level in the warm period.

High resistance to common and inexpensive antibiotics is observed among *Campylobacter* strains analyzed – erythromycin (92.8%), ampicillin (95.2%), ciprofloxacin (85.7%), tetracycline (92.8%), and gentamycin (7.1%). This finding raises concerns about the effectiveness of such antibiotics for the treatment of animal diseases. Therefore, improving awareness and understanding of antimicrobial resistance is an issue that must be addressed through the implementation of specific programs among professionals and farmers. In addition, a competent authority must strengthen the surveillance programs targeting antibiotic residues in poultry meat. Mandatory biosecurity measures (Moroccan Avian low 49-99) are considered essential to prevent flock colonization with *Campylobacter* spp.

## Authors’ Contributions

RA conceived the work and conducted the fieldwork with BK, HE, and KE. BB and RT designed and supervised the study. BB drafted the manuscript, interpreted data and RT and HK edited the manuscript. RT and BB reviewed the manuscript. All authors read and approved the final manuscript.
